# A cell-wall-modifying gene-dependent CLE26 peptide signaling confers drought resistance in *Arabidopsis*

**DOI:** 10.1093/pnasnexus/pgae049

**Published:** 2024-02-01

**Authors:** Satoshi Endo, Hiroo Fukuda

**Affiliations:** Department of Bioscience and Biotechnology, Kyoto University of Advanced Science, Kyoto 621-8555, Japan; Department of Bioscience and Biotechnology, Kyoto University of Advanced Science, Kyoto 621-8555, Japan; Akita Prefectural University, Akita 010-0195, Japan

**Keywords:** cell wall, peptide signaling, CLE, abiotic stress, drought

## Abstract

Plants respond to various environmental stimuli in sophisticated ways. Takahashi et al. (2018) revealed that CLAVATA3/EMBRYO SURROUNDING REIGON-related 25 (CLE25) peptide is produced in roots under drought stress and transported to shoots, where it induces abscisic acid biosynthesis, resulting in drought resistance in *Arabidopsis*. However, the drought-related function of the CLE26 peptide, which has the same amino acid sequence as CLE25 (except for one amino acid substitution), is still unknown. In this study, a phenotypic analysis of *Arabidopsis* plants under repetitive drought stress treatment indicates that *CLE26* is associated with drought stress memory and promotes survival rate at the second dehydration event. Additionally, we find that a loss-of-function mutant of a cell-wall-modifying gene, *XYLANASE1* (*XYN1*), exhibits improved resistance to drought, which is suppressed by the mutation of *CLE26*. *XYN1* is down-regulated in response to drought in wild-type plants. A further analysis shows that the synthetic CLE26 peptide is well transported in both *xyn1* and drought-pretreated wild-type plants but not in untreated wild-type plants. These results suggest a novel cell wall function in drought stress memory; short-term dehydration down-regulates *XYN1* in xylem cells, leading to probable cell wall modification, which alters CLE26 peptide transport, resulting in drought resistance under subsequent long-term dehydration.

**Significance Statement**

Plants have evolved various strategies to combat drought stress. One of these strategies involves the CLE25 peptide, which acts as a signaling molecule to transmit the drought stress response through the apoplastic space. In this study, we report that *CLE26*, with a previously unknown function in drought response, confers drought resistance, like *CLE25*, but only under drought stress memory. Transport of CLE26 peptide changes in plants previously subjected to drought stress. We further show that the function and transport of CLE26 depend on a cell-wall-modifying gene, *XYLANASE1*, whose expression level is likely regulated by drought stress memory. This study reveals a novel function of *CLE26* in drought stress memory and a possible regulation of CLE26 peptide signaling at the transportation level.

## Introduction

Plants evolved the vascular system to expand their territory in terrestrial environments. Vascular plants transport water and nutrients from roots to above-ground organs through the xylem. However, land plants are often exposed to severe stresses, including drought, induced by drastic environmental changes. To adapt to drought stress, plants have evolved various response systems, such as dehydration tolerance by alleviating osmotic and/or oxidative damage, and dehydration avoidance by regulating water uptake and water loss, all of which determine drought resistance (1–3).

Xylem mediates not only the distribution of water but also the long-distance transport of signals. Drought induces the expression of CLAVATA3/EMBRYO SURROUNDING REGION-related 25 (*CLE25*) in roots, and the synthesized CLE25 peptide is transported to shoots through the xylem, where the peptide promotes the biosynthesis of abscisic acid (ABA), a stress hormone (4). When *CLE2* and *CLE3* are up-regulated in roots, specific metabolic and defense responses are induced in shoots, respectively (5, 6). Okamoto et al. (7) identified a native CLE2 peptide in the xylem exudate, suggesting that these peptides function in specific cellular events as long-distance signals via xylem transport. Our previous study suggested the possibility that xylem transport is affected by plant cell walls that have been modified in response to various environmental stimuli (8). However, it remains unknown whether plant cell wall alterations are involved in drought response. We previously generated ∼50 types of transgenic *Arabidopsis* lines expressing different xylem cell-wall-related genes under the control of the xylem-specific *Arabidopsis Tracheary Element Differentiation-related 4* promoter (9). These transgenic lines displayed alterations in xylem cell walls but did not exhibit any large changes in plant growth. Using a hypocotyl-to-leaf fluorescein transport assay that enables a rapid observation of xylem transport, we found that the xylem transport pattern of fluorescein was affected in a cargo-dependent manner in the transgenic lines (8). The fluorescence of 5(6)-carboxyfluorescein (FAM)-labeled CLE25 peptide (CLE25F) was relatively weak in leaf veins, whereas that of CLE26F was strong. CLE26 had the same amino acid sequence as CLE25, except for one amino acid substitution, and did not induce drought response in wild-type (WT) plants (4). Interestingly, the fluorescence from FAM-labeled CLE peptides was altered in the T19 transgenic lines overexpressing *Arabidopsis XYLANASE1* (*XYN1*), which encoded a glycosyl hydrolase family 10 protein with xylanase and/or xylan endotransglycosylase activity (8). This alteration was observed only in the T19 lines among all transgenic lines tested. These results suggest that cell-wall-related and/or cargo-dependent xylem transport might be involved in plant drought response.

Therefore, in this study, we investigated the relationship between *XYN1* and drought stress, considering a possible involvement of xylem transport regulation of CLE25 and CLE26 peptides. The results revealed a stress memory, according to which short-term dehydration down-regulates *XYN1* expression, which allows the activation of CLE26 peptide signaling, resulting in greater drought resistance under subsequent long-term dehydration.

## Results

### *XYN1*-dependent drought resistance

**Fig. 1.**
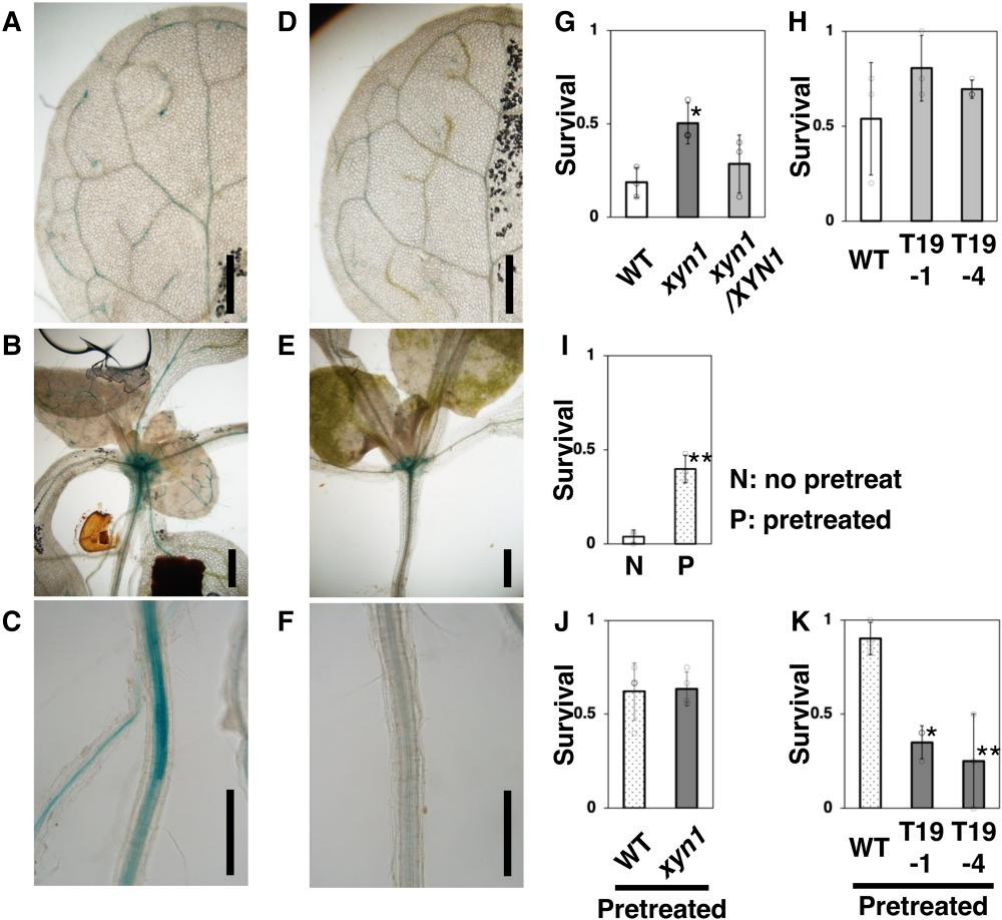
*XYN1* expression and *XYN1*-associated drought resistance in *A. thaliana*. a–f) *XYN1* expression upon dehydration. X-Gluc staining of plants harboring *pXYN1::GUS* before (a–c) or after 2 days of dehydration (d–f). Close-up views of maturing leaves (a, d), hypocotyls with juvenile leaves (b, e), and roots close to the hypocotyls (c, f). Scale bars, 0.5 mm. g–k) Drought survival rates of the *xyn1* mutant (g, j), *xyn1/XYN1* complementation (g), T19 lines (h, k), and the drought-pretreated WT (i). In (i–k), plants had been drought-pretreated (P or pretreated). Values are presented as the mean ± SD (*n* = 3 [g, h, i, k]; *n* = 4 [j], open circles). In (g, i, k), the asterisks indicate significant differences compared with WT (*P < 0.05, **P < 0.01; Dunnett's test [g, k]; Welch's *t*-test [i]).

To understand the relationship between *XYN1* and drought stress, we first determined the expression pattern of *XYN1* under normal conditions and then examined whether its expression is altered in response to drought stress. To perform this experiment, a *pXYN1::GUS* construct was introduced into the WT. The XYN1::GUS signal was detected specifically in xylem cells of leaves and roots (Fig. 1a–c), as previously reported by Suzuki et al. (10). Dehydration diminished the pXYN1::GUS signal in both leaves and roots (Fig. 1d–f). A quantification of *XYN1* mRNA levels confirmed a conspicuous decrease in *XYN1* expression in response to dehydration (Fig. S2a). This result suggests that XYN1 is potentially involved in the drought stress response in plants.

Next, we examined the dehydration resistance of three *Arabidopsis* genotypes: loss-of-function *xyn1* mutant; *xyn1* complementation line carrying a genomic fragment of *XYN1* (*xyn1/XYN1*); and two T19 lines (T19-1 and T19-4) overexpressing *XYN1* under the control of a xylem-specific promoter. Each genotype was treated with soil dehydration for 7 days. The *xyn1* mutant, but not *xyn1/XYN1*, showed a higher survival rate than WT plants under dehydration stress (Figs. 1g and S2b). On the contrary, both T19 lines exhibited no significant change in survival rate (Fig. 1h). This result indicates that the loss-of-function mutation of *XYN1* increases drought resistance.

Plant responses to drought stress vary depending on many factors, such as the intensity and duration of the stress and plant genotype and growth phase (11). Interestingly, plants have stress memory, i.e. an imprint of the previous stress episodes (12–16). This imprint/stress memory is defined as a collection of the structural, genetic, and biochemical modifications that occurred as a consequence of stress exposure, making the plant more resistant to future exposure to the same stress factor. To determine whether stress memory was established under our experimental conditions, WT plants were pretreated with a 2-day dehydration period, followed by 3 days of rehydration, and then subjected to >6 days of dehydration. Drought resistance increased in WT plants that had been pretreated (Figs. 1i and S2c), suggesting the presence of stress memory. Next, we compared the pretreated WT plants with *xyn1* mutant plants. The drought survival rate of the *xyn1* mutant plants was similar to that of the pretreated WT plants (Fig. 1j). In contrast, the pretreatment did not enhance the drought resistance of T19-1 and T19-4 lines (Fig. 1k). Thus, together with the finding that drought stress reduces *XYN1* expression, these results suggest that the down-regulation of *XYN1* may be required for drought stress memory.

### *CLE26*-mediated drought resistance

**Fig. 2.**
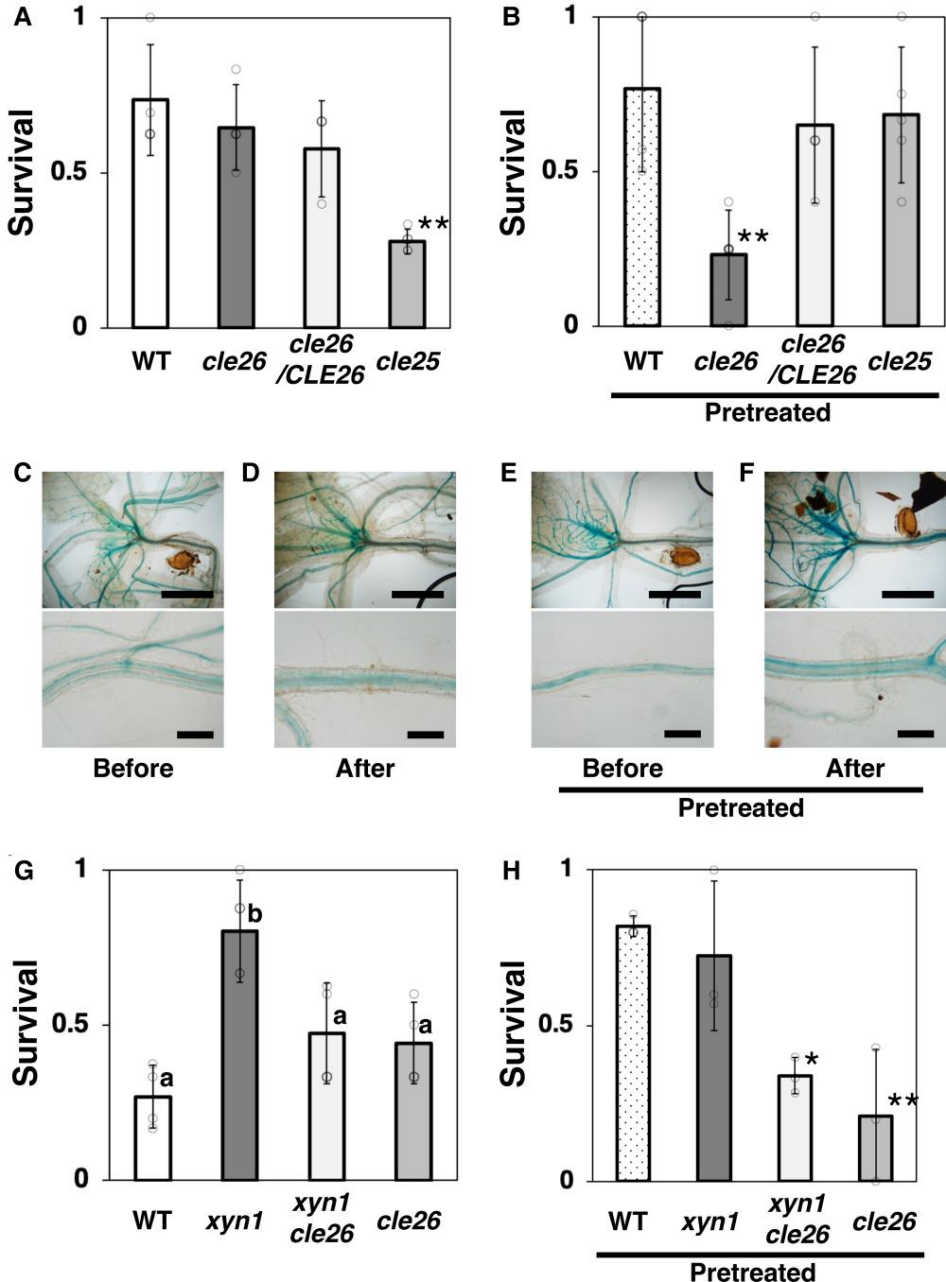
*CLE26*-mediated drought resistance in *Arabidopsis*. a and b) Drought survival rates of WT, the *cle26* mutant, *cle26/CLE26* complementation, and the *cle25* mutant plants without the pretreatment (a) or with the pretreatment (b). Values are presented as the mean ± SD (*n* = 3–5; open circles). Asterisks indicate significant difference compared with the WT (**P < 0.01; Dunnett's test). c–f) *CLE26* expression upon dehydration. Short (3 h) X-Gluc staining of plants harboring *pCLE26::GUS* before dehydration (c, e) and after 1 day of dehydration (d, f) without the pretreatment (c, d) or with the pretreatment (e, f). Scale bars: 1 mm (upper panel), 200 µm (lower panel). g and h) Drought survival rates of WT, the *xyn1* mutant, the *xyn1 cle26* double mutant, and the *cle26* mutant plants without the pretreatment (g) or with the pretreatment (h). Values are presented as the mean ± SD (*n* = 4 [g]; *n* = 3 [h], open circles). Different letters in (g) indicate significant differences (*P* < 0.05; Tukey–Kramer test). Asterisks in (h) indicate significant differences compared with the WT (**P < 0.01, *P < 0.05; Dunnett's test).

Takahashi et al. (4) reported that drought stress induces *CLE25* expression in roots. The root-derived CLE25 peptide moves from roots to leaves, where it mediates the expression of osmotic stress- and ABA-inducible genes, resulting in drought resistance. In contrast, the CLE26 peptide, which shows only one amino acid substitution compared with CLE25 peptide and exhibits the same biological function in root growth as the CLE25 peptide (17, 18), does not function like CLE25 in drought resistance (4). Our experiment using loss-of-function mutants of *CLE25* and *CLE26* (*cle25-cr1* and *cle26-cr1*: Yamaguchi et al. (19)) confirmed that *CLE25*, but not *CLE26*, was required for drought resistance (Fig. 2a). Next, we examined the involvement of *CLE25* and *CLE26* in drought stress memory. After the drought pretreatment, the survival rate of *cle25* plants was similar to that of WT plants, whereas that of *cle26* plants was significantly reduced (Fig. 2b). This *cle26* phenotype was complemented by the introduction of the *CLE26* genomic fragment, suggesting that *CLE26* is required for drought stress memory. *CLE25* is transcriptionally up-regulated in roots in response to dehydration (4). Therefore, we generated transgenic plants harboring the *pCLE26::GUS* construct, in which 3 kb upstream and 3 kb downstream sequences of *CLE26* had been integrated, and investigated whether *CLE26* expression is regulated by drought stress. The pCLE26::GUS signal was observed in vascular bundles, as previously reported (20). Drought stress, with or without the drought pretreatment, showed no clear change in its expression level or pattern in whole plants (Figs. 2c–f and S3), implying that the potential function of *CLE26* in drought response is not likely under transcriptional regulation.

To investigate the involvement of *CLE26* in *XYN1*-dependent drought resistance, we generated the *xyn1 cle26* double mutant. The enhanced survival rate of pretreated *xyn1* plants was suppressed by the mutation of *CLE26*, and the survival rate of the *xyn1 cle26* double mutant was similar to that of the *cle26* single mutant and the WT (Fig. 2g). On the contrary, the mutation of *CLE26* suppressed the pretreatment-dependent enhancement of drought resistance in both WT and *xyn1* plants (Fig. 2h). These results suggest that CLE26 peptide signaling is involved in *xyn1*-dependent drought resistance.

**Fig. 3.**
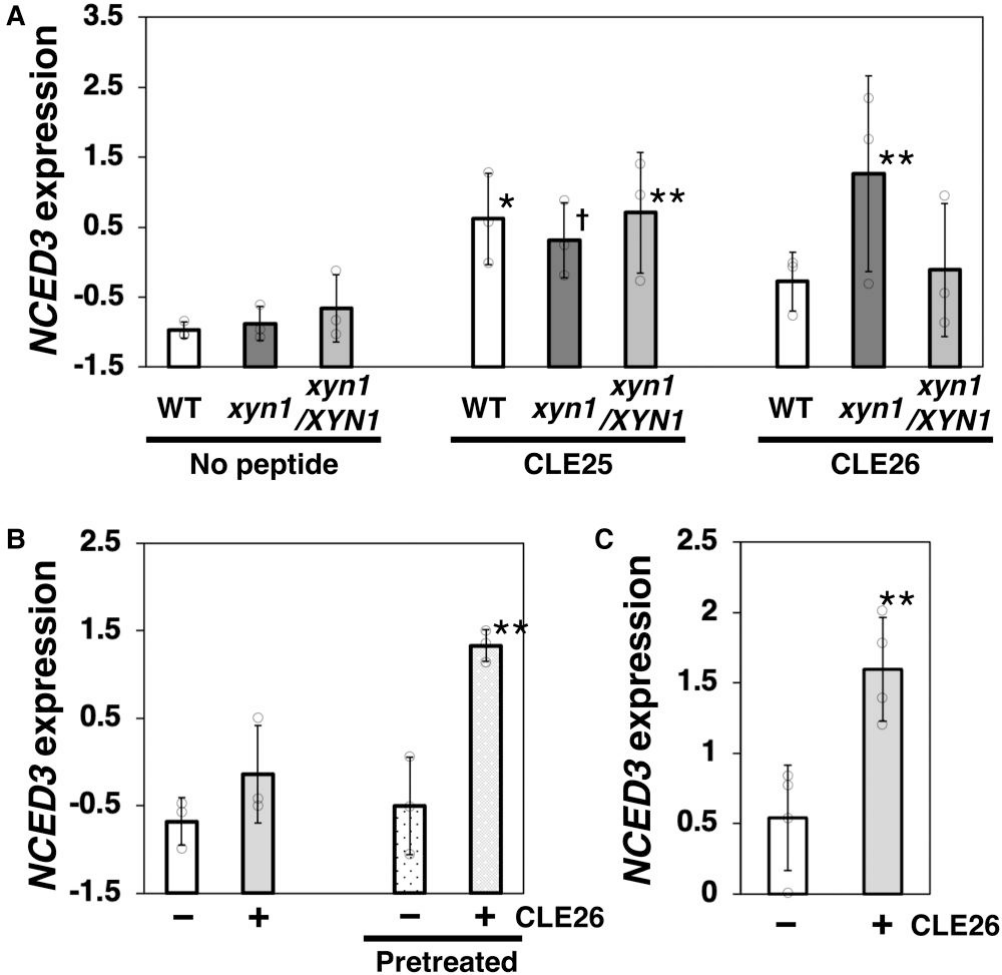
CLE26-peptide-mediated drought response in *Arabidopsis*. Induction of *NCED3* expression in WT, the *xyn1* mutant, and *xyn1/XYN1* complementation plants by the CLE26 peptide. Water-only, CLE25, or CLE26 synthetic peptide was applied to roots (a, b) or leaves (c) prior to the sampling of shoots. In (b, c), only WT plants were examined and the indicated ones had been drought pretreated (pretreated). Each replicate was normalized to an average of 0 and an SD of 1. Values are presented as the mean ± SD (*n* = 3 [a, b]; *n* = 4 [c], open circles). Asterisks and a dagger in (a, b) indicate significant and nearly significant differences compared with the WT with no peptide treatment (**P < 0.01, *P < 0.05, **^†^***P* = 0.12; Dunnett's test). Asterisks in (c) indicate significant differences compared with the no-peptide treatment (**P < 0.01; Welch's *t*-test).

CLE25 peptide acts as a long-distance signal from roots to shoots, inducing drought resistance in *Arabidopsis* (4). ABA is primarily synthesized in leaf vascular tissues through the dehydration-inducible expression of *NINE-CIS-EPOXYCAROTENOID DIOXYGENASE 3* (*NCED3*), which encodes a key ABA biosynthetic enzyme (21). Application of the synthesized CLE25 peptide, but not CLE26 peptide, to roots induces *NCED3* expression in shoots (4). In this study, the application of the CLE25 peptide enhanced *NCED3* expression in *xyn1*, *xyn1/XYN1*, and WT plants (Fig. 3a). The CLE26 peptide did not induce *NCED3* expression in WT plants, as previously shown. However, in the *xyn1* background, root treatment with the CLE26 peptide induced *NCED3* expression in shoots (Fig. 3a). The *xyn1/XYN1* complementation line did not show CLE26-peptide-dependent *NCED3* induction. As aforementioned, the drought pretreatment enhances drought resistance in a *CLE26*-dependent manner. Therefore, we examined the inducibility of *NCED3* in the shoots of drought-pretreated WT plants following root treatment with the CLE26 peptide. As expected, the CLE26 peptide strongly induced *NCED3* expression in the pretreated plants (Fig. 3b). In addition, we observed that CLE26 peptide application to shoots was able to induce *NCED3* expression in untreated WT plants (Fig. 3c). These results suggest that the drought pretreatment enhances the movement of the CLE26 peptide, acting in shoots.

### *XYN1*-associated CLE26 peptide transport

To investigate the *XYN1-*dependent alteration of xylem transport in detail, we observed the accumulation of fluorescein in the leaf veins of *xyn1* and T19 plants (Fig. S4) using the hypocotyl-to-leaf fluorescein assay (8). A fluorescent signal in leaf veins was more intense in T19 than in WT plants (Fig. S4a and b), confirming our previous result (8). In contrast, the fluorescein signal was weaker in *xyn1* than in WT leaf veins (Fig. S4c and d). These results indicate that the opposite xylem transport phenotypes of T19 and *xyn1* plants result from the up- and down-regulation of *XYN1* expression, respectively.

**Fig. 4.**
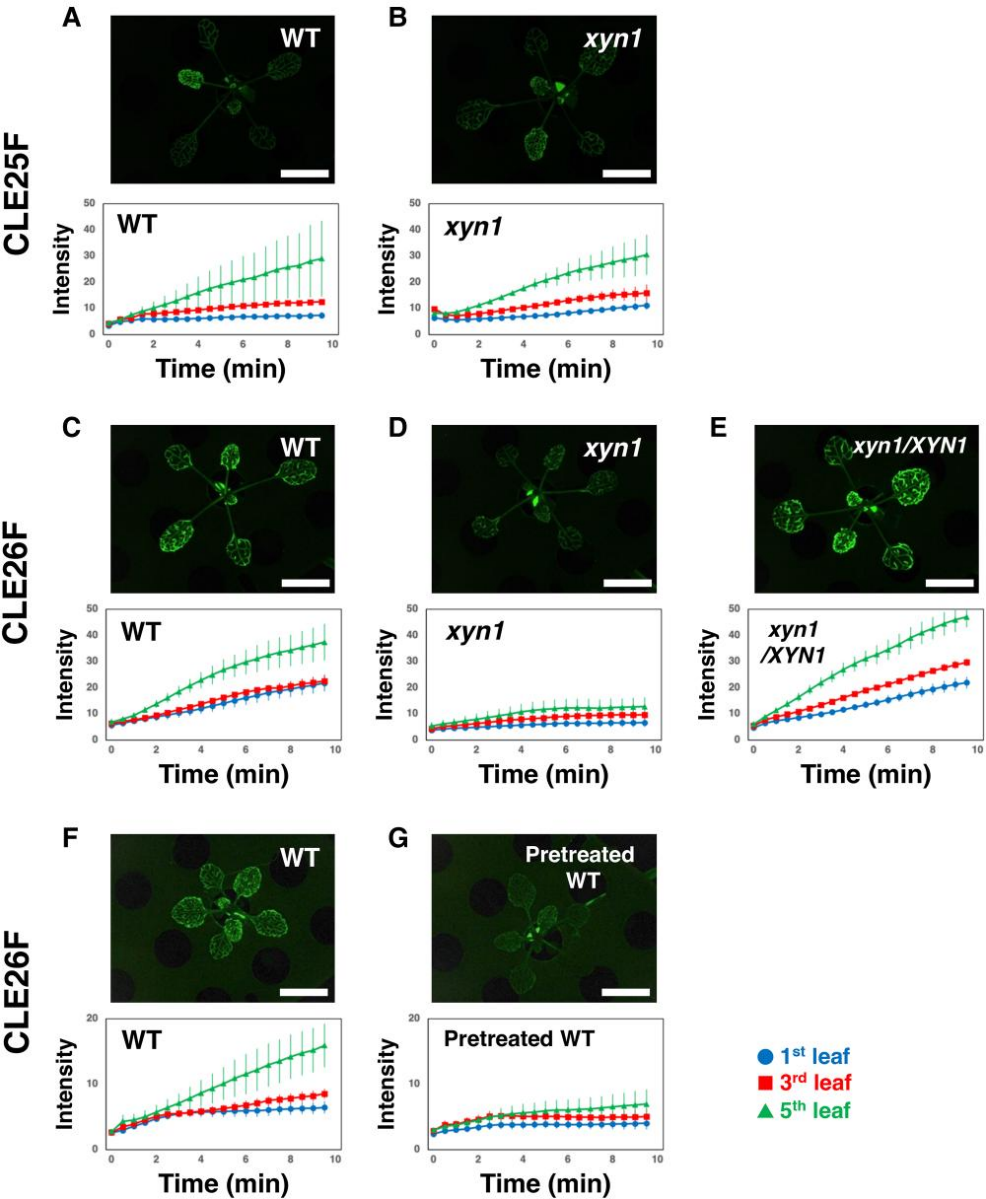
*XYN1*-associated distribution of the fluorescent-labeled CLE26 peptide in *Arabidopsis* at 10 min after application. a and b) CLE25F fluorescence in the WT and the *xyn1* mutant plants. c–e) CLE26F fluorescence in WT, the *xyn1* mutant, and *xyn1/XYN1* complementation plants. f, g) CLE26F fluorescence in WT without the pretreatment (f) or with the pretreatment (g). Upper panels show fluorescence images of the indicated lines at 10 min after the application of FAM-labeled CLE25 (CLE25F) (a, b) and CLE26F (c–g) to the cut ends of hypocotyls. Images were taken from the top of plants with six leaves, as described in Ref. (8). Scale bars, 5 mm. Lower panels show the time-lapse analysis of CLE25F (a, b) and CLE26F (c–g) accumulation in leaves. Changes in fluorescence intensity (arbitrary unit) in the first (circles), third (squares), and fifth (triangles) leaves are shown at the indicated time points after peptide application. Leaves are numbered from adult to young. Values are presented as the mean of three observations ± SEM (*n* = 3).

Our previous study indicated a difference in fluorescence accumulation between FAM-labeled peptides, CLE25F and CLE26F, in leaf veins (8). Similarly, the application of CLE25F at hypocotyl cut ends produced a relatively weak vein fluorescence in WT as well as *xyn1* plants (Fig. 4a and b). In contrast, CLE26F application resulted in a relatively intense vein fluorescence in WT plants but weak fluorescence in *xyn1*, which was restored in *xyn1/XYN1* plants (Fig. 4c–e). Similarly, the drought pretreatment reduced CLE26F fluorescence in WT plants (Fig. 4f and g). These results clearly indicate a tight correlation between a weak CLE fluorescence signal in leaf veins and high drought resistance. This is also supported by shoot-specific *NCED3* induction experiments, in which root-applied CLE26 peptide induced shoot-specific *NCED3* expression in *xyn1* and drought-pretreated WT plants (Fig. 3a and b).

**Fig. 5.**
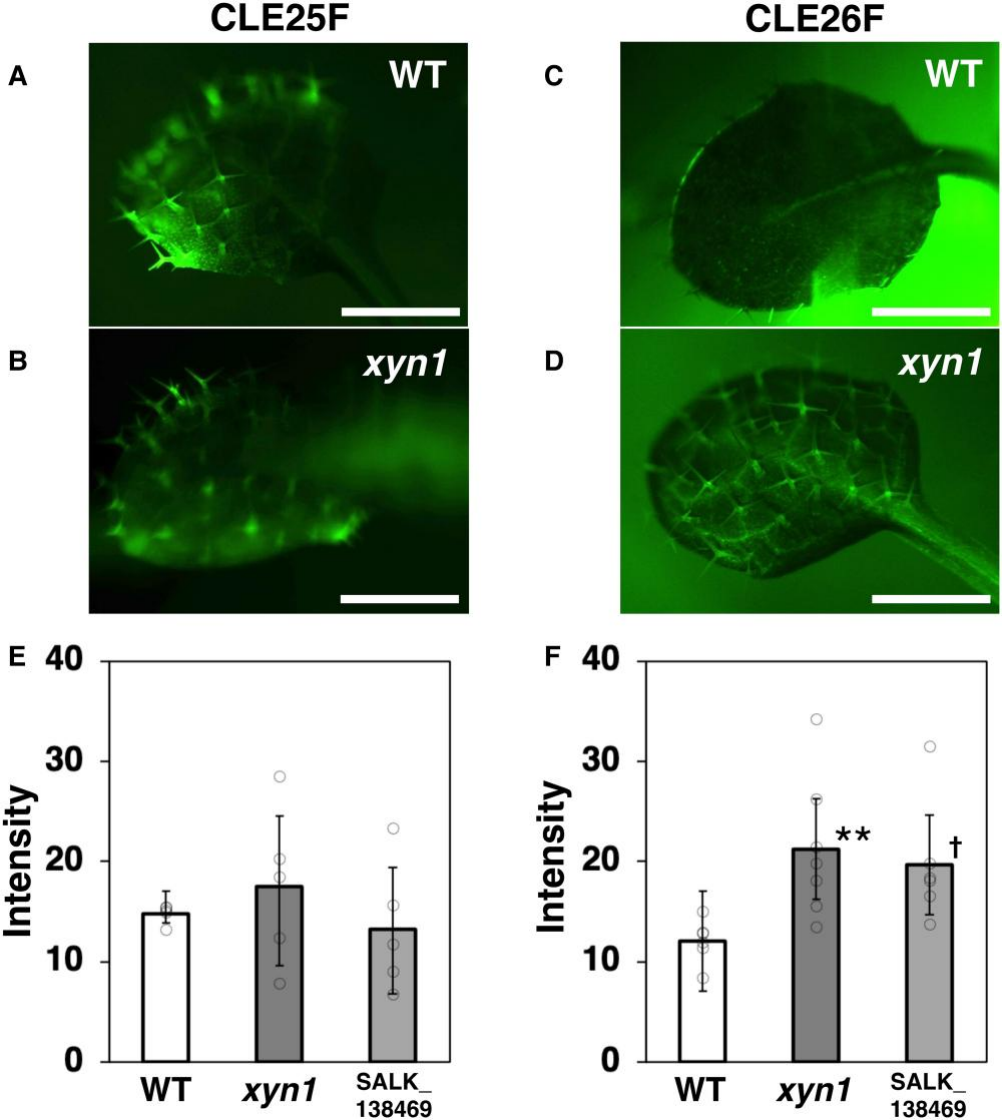
*XYN1*-associated distribution of the fluorescent-labeled CLE peptides in *Arabidopsis*, at 1 h after application. a–d) Fluorescence images of the WT, the *xyn1* mutant plants at 1 h after the application of CLE25F (a, b) or CLE26F (c, d) to the cut ends of hypocotyls. Scale bars, 5 mm. e, f) Changes in fluorescence intensity (arbitrary unit) in the third leaves of WT, the *xyn1* mutant, and another *xyn1* mutant (SALK_138469) plants at 1 h after application. Values are presented as the mean ± SD (*n* = 5 [e]; *n* = 6 or 7 [f], open circles). Asterisks and a dagger indicate significant and nearly significant differences compared with the WT (**P < 0.01, **^†^***P* = 0.06; Dunnett's test).

We speculated that the weak CLE-FAM fluorescence signal in leaf veins implies active xylem transport beyond veins in leaves, and conversely, the high intensity of vein fluorescence reflects inactive transport from xylem in leaves, resulting in fluorescence within veins. To detect fluorescence signals spread beyond leaf veins, we used two different methods. First, we observed the fluorescein signal in detached leaves immediately after the transport assay in a high pH solution (Fig. S5) because the fluorescence of fluorescein drastically reduces in acidic pH; importantly, apoplastic pH is acidic in plants (22, 23). The *xyn1* and pretreated WT leaves showed higher CLE26F fluorescence than WT leaves (Fig. S5a–f). In contrast, T19 leaves showed lower CLE26F fluorescence (Fig. S5g–i). Second, we performed a diffusion assay to visualize fluorescence over leaf veins by applying a tracer to young seedlings for 1 h. In this assay, CLE25F fluorescence spread and reached the trichomes in both WT and *xyn1* plants (Fig. 5a, b, and e). On the contrary, the CLE26F signal did not spread to trichomes in WT plants, but it did in *xyn1* (Fig. 5c, d, and f), which is consistent with the *NCED3*-inducing activity of CLE25 and CLE26 peptides (Fig. 3a). These results strongly suggest that the *XYN1*-dependent regulation of CLE26 peptide signaling occurs at the transition of the CLE26 peptide from leaf veins to whole leaves.

## Discussion

### *XYN1*-dependent transport of CLE26 peptide

Signaling molecules, as well as water and nutrients, are transported via xylem and released into the extracellular space. In this study, we revealed that under repetitive drought stress, the CLE26 peptide is transported and functions in inducing drought resistance.

Our previous study indicated that xylem-specific expression or suppression of some cell-wall-related genes alters cargo transport patterns in leaves (8). We here revealed that suppression of *XYN1* enhances the release of CLE26 peptide from leaf veins, while the presence of *XYN1* retains the CLE26 peptide inside the leaf veins. Plant cell walls can affect the diffusion and concentration of apoplastic molecules by functioning as a permeability barrier and an ion-exchange resin-like reservoir (24, 25). Indeed, Zhu et al. (26) reported that the cell wall contributes to iron supply to alleviate iron-deficiency symptoms in plants. Plants have complicated cell wall structures, which are frequently modified in response to environmental cues and developmental signals by a large number of cell-wall-modifying genes, the biological roles of most of which remain unknown (27, 28). In this study, we show that *XYN1* plays an important role in drought resistance through the modification of xylem transport. Future studies on cell wall dynamics may provide keys to understanding the unique strategies employed by plants to respond to changing environments.

### *XYN1*-dependent drought resistance

Maize *WI5*, encoding an endo-1,4-β-xylanase, is required for water transport through xylem (29). Furthermore, a loss-of-function mutant of *OsXYN1*, a rice homolog of *W15*, shows a defect in drought resistance (30). These facts suggest the importance of xylanase-associated structural decoration of cell walls under the drought stress condition. We found that the *xyn1* mutant exhibited increased drought resistance. On the contrary, *XYN1* was immediately down-regulated when soil was dehydrated in pretreatment. *XYN1*, which encodes a protein exhibiting a xylanase and/or a xylan endotransglycosylase activity (10, 31), is preferentially expressed in the xylem. Because *XYN1* down-regulation continues at least for >3 days after rehydration, xylem cell walls should be formed without XYN1 during the rehydration period prior to the next drought. This modified cell wall formation may result in resistance to the second drought treatment after rehydration, which is similar to the resistance of *xyn1* plants to drought without the pretreatment (Fig. 1). Indeed, down-regulation of aspen *PtxtXyn10A*, the closest homolog of *Arabidopsis XYN1*, causes various changes in cell wall structure, including cellulose microfibril arrangement (31). On the contrary, xylem-specific *XYN1* overexpression altered secondary cell wall structure, resulting in reduced cell wall recalcitrance in *Arabidopsis* (8).

Preexposure to stress often alters the subsequent responses of plants by producing faster and/or stronger reactions, implying that plants exercise a form of stress memory. Stress memory is involved in modifications at different levels, including morphological, physiological, transcriptional, translational, and epigenetic (12–16). During recurring dehydration stresses, *Arabidopsis* plants demonstrate transcriptional memory of the stress, as displayed by the increased rates of transcription and elevated transcript levels of a subset of stress-responsive genes. Plants also display drought stress memory on the physiological and biochemical levels, which allow changes in photosynthetic rates, phytohormone levels, or plant biomass (16). This study revealed a novel drought stress memory system in which drought memory is written in cell walls as an alteration of cell wall structure, which allows the transport of a stress signal from leaf veins to whole leaves at the second drought stress event.

### *CLE26*-mediated drought resistance

Two similar CLE peptides, CLE25 and CLE26, perform the same function in many plant developmental processes, including root growth (17), xylem development (18), and phloem development (32, 33). In drought response, however, these two peptides behave differently (4). Drought stress induces only *CLE25* in roots, and the CLE25 peptide is transported to shoots via xylem and induces ABA biosynthesis. In this study, we found that *CLE26* also functions in drought resistance by drought stress memory, i.e. by enhancing drought stress in plants that had previously been exposed to short-term dehydration. Previous studies indicated that *CLE26* does not show any significant transcriptional up-regulation in response to dehydration (34). Our pCLE26::GUS reporter analysis confirmed this result. Furthermore, when supplied exogenously to roots, the CLE25 peptide, but not CLE26 peptide, induced *NCED3* expression in shoots. Once drought pretreated, however, the application of CLE26 peptide to roots induced *NCED3* expression in shoots. Indeed, when *XYN1* was down-regulated by the drought pretreatment, CLE26F, which was previously retained within leaf veins, diffused beyond leaf veins and even reached trichomes. These results strongly suggest that CLE26 peptide signaling functions as a component of drought memory in leaves.

In conclusion, we investigated the involvement of *XYN1* in the modification of the transport and function of CLE26 peptide in drought resistance, and successfully indicated a novel drought stress memory, in which short-term dehydration down-regulates *XYN1* expression, which promotes CLE26 peptide transport in leaves, resulting in higher drought resistance at the subsequent long-term dehydration event.

## Materials and methods

### Plant materials and growth conditions

*Arabidopsis thaliana* (L.) Heynh. accession Columbia (Col-0; WT), *xyn1* loss-of-function mutants (SALK_132155 and SALK_138469), T19 lines overexpressing *XYN1* (8), and *cle25* and *cle26* loss-of-function mutants (*cle25-cr1* and *cle26-cr1*, respectively (19)) were used in this study.

Plants were grown under long-day (LoD; 16 h day/8 h night) or short-day (ShD; 10 h day/14 h night) photoperiod at 22–23 °C either on vermiculite (VS kakou) with diluted Hyponex (Hyponex Japan) at relative humidity (RH) >45% or on half-strength Murashige and Skoog (1/2 MS) agar medium containing sucrose. Illumination during the subjective day was provided using LED or fluorescent white light bulbs at 40–80 µmol m^2^ s^−1^.

### Vector construction

Primers used for vector construction are shown in Table S1. To generate *xyn1/XYN1* and *cle26/CLE26* complementation lines, genomic DNA fragments of *XYN1* and *CLE26*, respectively, spanning a 3-kb sequence upstream of the start codon, coding region, and 3-kb sequence downstream of the stop codon of the corresponding gene were cloned into pBG (35). The resultant plasmids were further converted into *pXYN1::GUS* and *pCLE26::GUS* by swapping the coding region of *XYN1* and *CLE26*, respectively, with the *GUS* gene. *Arabidopsis* plants were transformed by floral dip with GV3101 (pMP90) containing one of the above-mentioned constructs.

### Histology

Plants harboring *pXYN1::GUS* or *pCLE26::GUS* were treated with ice-cold 90% acetone for a few days, incubated in X-Gluc solution (2 mM X-Gluc, 0.5 mM potassium ferricyanide, 0.5 mM potassium ferrocyanide, 0.1 M sodium phosphate buffer [pH 7.4]) for 3 h (Fig. 2c–f) or 1 day (Fig. S3) at room temperature, and transferred to a clearing solution (8 g of chloral hydrate, 1 mL of glycerol, and 2 mL of water) on glass slides. Images were taken using BX51 (Olympus).

### Quantitative PCR

Total RNA was extracted from shoots or leaves using RNeasy Plant Mini (Qiagen), and reverse-transcribed into cDNA using SuperScript III (Invitrogen). Quantitative real-time PCR was performed on LightCycler 480 using TaqMan probe system (Roche). Primers used for real-time PCR are shown in Table S1. *UBQ10* was used as a reference gene.

### Drought treatment

To examine *XYN1* expression level, plants that had been grown for 2 weeks on 1/2 MS-agar under the ShD condition were placed on water-soaked filter paper for 16 h. Plants were sampled before and after the 3-h dehydration on dried filter paper. The *XYN1* expression level was analyzed by qPCR.

To perform the drought survival test, plants that had been grown until the four-leaf stage (*cle25*) or six-leaf stage (other genotypes) on vermiculite under the LoD condition were dehydrated on paper towels. After 1 day, RH was adjusted between 20 and 40%. Plants usually wilted in 7 or 8 days, while the comparison between WT plants with or without 8 or 9 days of pretreatment (Figs. 1i and S2c). Then, plants were rehydrated and returned to their normal condition. The number of surviving seedlings was counted (Fig. S1).

### Pretreatment

Plants at the four-leaf stage were dehydrated on paper towels for 1 day under the normal growth condition, and then transferred to the dry condition (RH = 20–40%) for 1 day. Subsequently, plants were rehydrated, and grown under normal conditions for 3 days.

### *NCED3* induction assay

Synthetic CLE peptides used for this assay are shown in Table S2. Plants were grown on 1/2 MS-agar under ShD at ∼90° to allow the roots to elongate on the surface of the agar medium and to keep shoots away from the surface. Then, 30 µL of 1 µM CLE25 peptide, 10 µM CLE26 peptide, or water was applied to the lower parts of roots (Fig. 3a) or leaves (Fig. 3c) of several plants of each genotype. Shoots were sampled after 6 h of incubation under the same growth conditions. To perform the assay with plants grown on vermiculite under LoD, 3 mL of 1 µM CLE26 peptide was added to each pot (Fig. 3b), when the plants had five leaves. Leaves were sampled after 2 days. *NCED3* expression was quantified by qPCR.

### Tracer assay

The FAM-labeled CLE peptides used for this assay are shown in Table S2. Mixtures of fluorescein, CLE25F, and CLE26F with rhodamine B (0.5 mM each) are used in Figs. 4 and S4. Diluted mixtures (0.1 mM each) and single CLE-FAM (0.2 mM) are used in Figs. S5 and 5, respectively. Plants were grown under the ShD-vermiculite condition (Figs. 4, S4, and S5) or the LoD-agar condition (Fig. 5), except the plants shown in Fig. 4f and g, which were grown and pretreated under the LoD-vermiculite condition. Tracers were applied to the cut ends of hypocotyls of plants at the six-leaf stage (Figs. 4, S4, and S5) or four-leaf stage (Fig. 5). During the 1 h of tracer application, plants were incubated at high RH. The fluorescence of fluorescein, CLE25F, or CLE26F was measured in the first, third, and fifth leaves (Figs. 4 and S4), the third and fourth leaves (Fig. S5), or the third leaves (Fig. 5). Images were taken using M205FA (Leica Microsystems).

## Supplementary Material

pgae049_Supplementary_Data

## Data Availability

The authors confirm that the data supporting the findings in this study are all included in the article and its supplementary material.

## References

[1] Blum A, Tuberosa R. 2018. Dehydration survival of crop plants and its measurement. J Exp Bot. 69:975–981. 29325054 10.1093/jxb/erx445 PMC6018961

[2] Gupta A, Rico-Medina A, Caño-Delgado AI. 2020. The physiology of plant responses to drought. Science. 368:266–269. 32299946 10.1126/science.aaz7614

[3] Yao T, et al 2021. Transcriptional regulation of drought response in Arabidopsis and woody plants. Front Plant Sci. 11:572137. 33488639 10.3389/fpls.2020.572137 PMC7820124

[4] Takahashi F, et al 2018. A small peptide modulates stomatal control via abscisic acid in long-distance signalling. Nature. 556:235–238. 29618812 10.1038/s41586-018-0009-2

[5] Ma D, Endo S, Betsuyaku S, Shimotohno A, Fukuda H. 2020. *CLE2* regulates light-dependent carbohydrate metabolism in Arabidopsis shoots. Plant Mol Biol. 104:561–574. 32980951 10.1007/s11103-020-01059-y

[6] Ma D, et al 2022. Root-specific *CLE3* expression is required for *WRKY33* activation in Arabidopsis shoots. Plant Mol Biol. 108:225–239. 35038066 10.1007/s11103-021-01234-9

[7] Okamoto S, Kawasaki A, Makino Y, Ishida T, Sawa S. 2022. Long-distance translocation of CLAVATA3/ESR-related 2 peptide and its positive effect on root sucrose status. Plant Physiol. 189:2357–2367. 35567530 10.1093/plphys/kiac227 PMC9342984

[8] Endo S, Iwai Y, Fukuda H. 2019. Cargo-dependent and cell wall-associated xylem transport in *Arabidopsis*. New Phytol. 222:159–170. 30317651 10.1111/nph.15540

[9] Endo S, Iwamoto K, Fukuda H. 2018. Overexpression and cosuppression of xylem-related genes in an early xylem differentiation stage-specific manner by the AtTED4 promoter. Plant Biotechnol J. 16:451–458. 28664596 10.1111/pbi.12784 PMC5787829

[10] Suzuki M, Kato A, Nagata N, Komeda Y. 2002. A xylanase, AtXyn1, is predominantly expressed in vascular bundles, and four putative xylanase genes were identified in the *Arabidopsis thaliana* genome. Plant Cell Physiol. 43:759–767. 12154138 10.1093/pcp/pcf088

[11] Harb A, Krishnan A, Ambavaram MMR, Pereira A. 2010. Molecular and physiological analysis of drought stress in Arabidopsis reveals early responses leading to acclimation in plant growth. Plant Physiol. 154:1254–1271. 20807999 10.1104/pp.110.161752 PMC2971604

[12] Kinoshita T, Seki M. 2014. Epigenetic memory for stress response and adaptation in plants. Plant Cell Physiol. 55:1859–1863. 25298421 10.1093/pcp/pcu125

[13] Fleta-Soriano E, Munné-Bosch S. 2016. Stress memory and the inevitable effects of drought: a physiological perspective. Front Plant Sci. 7:143. 26913046 10.3389/fpls.2016.00143 PMC4753297

[14] Crisp PA, Ganguly D, Eichten SR, Borevotz JO, Pogson BJ. 2016. Reconsidering plant memory: intersections between stress recovery, RNA turnover, and epigenetics. Sci Adv. 2:e1501340. 26989783 10.1126/sciadv.1501340 PMC4788475

[15] Oberkofler V, Pratx L, Bäurle I. 2021. Epigenetic regulation of abiotic stress memory: maintaining the good things while they last. Curr Opin Plant Biol. 61:102007. 33571730 10.1016/j.pbi.2021.102007 PMC8250047

[16] Liu X, Quan W, Bartels D. 2022. Stress memory responses and seed priming correlate with drought tolerance in plants: an overview. Planta. 255:45. 35066685 10.1007/s00425-022-03828-z PMC8784359

[17] Kinoshita A, et al 2007. Gain-of-function phenotypes of chemically synthetic CLAVATA3/ESR-related (CLE) peptides in *Arabidopsis thaliana* and *Oryza sativa*. Plant Cell Physiol. 48, 1821–1825. 17991631 10.1093/pcp/pcm154

[18] Kondo Y, Hirakawa Y, Kieber JJ, Fukuda H. 2011. CLE peptides can negatively regulate protoxylem vessel formation via cytokinin signaling. Plant Cell Physiol. 52:37–48. 20802224 10.1093/pcp/pcq129 PMC3023848

[19] Yamaguchi YL, et al 2017. A collection of mutants for CLE-peptide-encoding genes in *Arabidopsis* generated by CIRSPR/Cas9-mediated gene targeting. Plant Cell Physiol. 58:1848–1856. 29036337 10.1093/pcp/pcx139

[20] Jun J, et al 2010. Comprehensive analysis of *CLE* polypeptide signaling gene expression and overexpression activity in Arabidopsis. Plant Physiol. 154:1721–1736. 20884811 10.1104/pp.110.163683 PMC2996011

[21] Endo A, et al 2008. Drought induction of Arabidopsis 9-cis-eppxycarotenoid dioxygenase occurs in vascular parenchyma cells. Plant Physiol. 147:1984–1993. 18550687 10.1104/pp.108.116632 PMC2492653

[22] Sugita H, et al 2003. Effects of pH and dissolved ions on fluorescence intensity of sodium fluorescein. J Geotherm Res Soc Japan. 25:211–225.

[23] Gjetting KSK, Ytting CK, Schulz A, Fugisang AT. 2012. Live imaging of intra- and extracellular pH in plants using pHusion, a novel genetically encoded biosensor. J Exp Bot. 63:3207–3218. 22407646 10.1093/jxb/ers040 PMC3350929

[24] Kramer EM, Frazer NL, Baskin TI. 2007. Measurement of diffusion within the cell wall in living roots of *Arabidopsis thaliana*. J Exp Bot. 58:3005–3015. 17728296 10.1093/jxb/erm155

[25] Meychik N, Nikolaeva Y, Kushunina M. 2021. The significance of ion-exchange properties of plant root cell walls for nutrient and water uptake by plants. Plant Physiol Biochem. 166:140–147. 34107383 10.1016/j.plaphy.2021.05.048

[26] Zhu XF, Wang B, Song WF, Zhen SJ, Shen RF. 2016. Putrescine alleviates iron deficiency via NO-dependent reutilization of root cell-wall Fe in Arabidopsis. Plant Physiol. 170:558–567. 26578707 10.1104/pp.15.01617 PMC4704603

[27] Le Gall H, et al 2015. Cell wall metabolism in response to abiotic stress. Plants. 4:112–166. 27135320 10.3390/plants4010112 PMC4844334

[28] Houston K, Tucker MR, Chowdhury J, Shirley N, Little A. 2016. The plant cell wall: a complex and dynamic structure as revealed by the responses of genes under stress conditions. Front Plant Sci. 7:984. 27559336 10.3389/fpls.2016.00984 PMC4978735

[29] Hu X, et al 2020. Maize *WI5* encodes an endo-1,4-β-xylanase required for secondary cell wall synthesis and water transport in xylem. J Integr Plant Biol. 62:1607–1624. 32129568 10.1111/jipb.12923 PMC7587005

[30] Tu B, et al 2020. Membrane-associated xylanase-like protein OsXYN1 is required for normal cell wall deposition and plant development in rice. J Exp Bot. 71:4797–4811. 32337581 10.1093/jxb/eraa200

[31] Derba-Maceluch M, et al 2015. Suppression of xylan endotransglycosylase *PtxtXyn10A* affects cellulose microfibril angle in secondary wall in aspen wood. New Phytol. 205:666–681. 25307149 10.1111/nph.13099

[32] Hu C, et al 2021. A CLE-BAM-CIK signalling module controls root protophloem differentiation in Arabidopsis. New Phytol. 233:282–296. 34651321 10.1111/nph.17791

[33] Qian P, et al 2022. A Dof-CLE circuit controls phloem organization. Nat Plants. 8:817–827. 35817820 10.1038/s41477-022-01176-0

[34] Czyzewicz N, et al 2015. Modulation of *Arabidopsis* and monocot root architecture by CLAVATA3/EMBRYO SURROUNDING REGION 26 peptide. J Exp Bot. 66:5229–5243. 26188203 10.1093/jxb/erv360 PMC4526925

[35] Yamaguchi M, et al 2010. VND-INTERACTING2, a NAC domain transcription factor, negatively regulates xylem vessel formation in *Arabidopsis*. Plant Cell. 22:1249–1263. 20388856 10.1105/tpc.108.064048 PMC2879754

