# Supplementary material for: A cell-wall-modifying gene-dependent CLE26 peptide signaling confers drought resistance in Arabidopsis
Source: PNAS Nexus. 2024 Feb 1;3(2):pgae049. doi: 10.1093/pnasnexus/pgae049 (PMC10863546; doi:10.1093/pnasnexus/pgae049)
Supplement: pgae049_Supplementary_Data [file pgae049_supplementary_data.pdf]

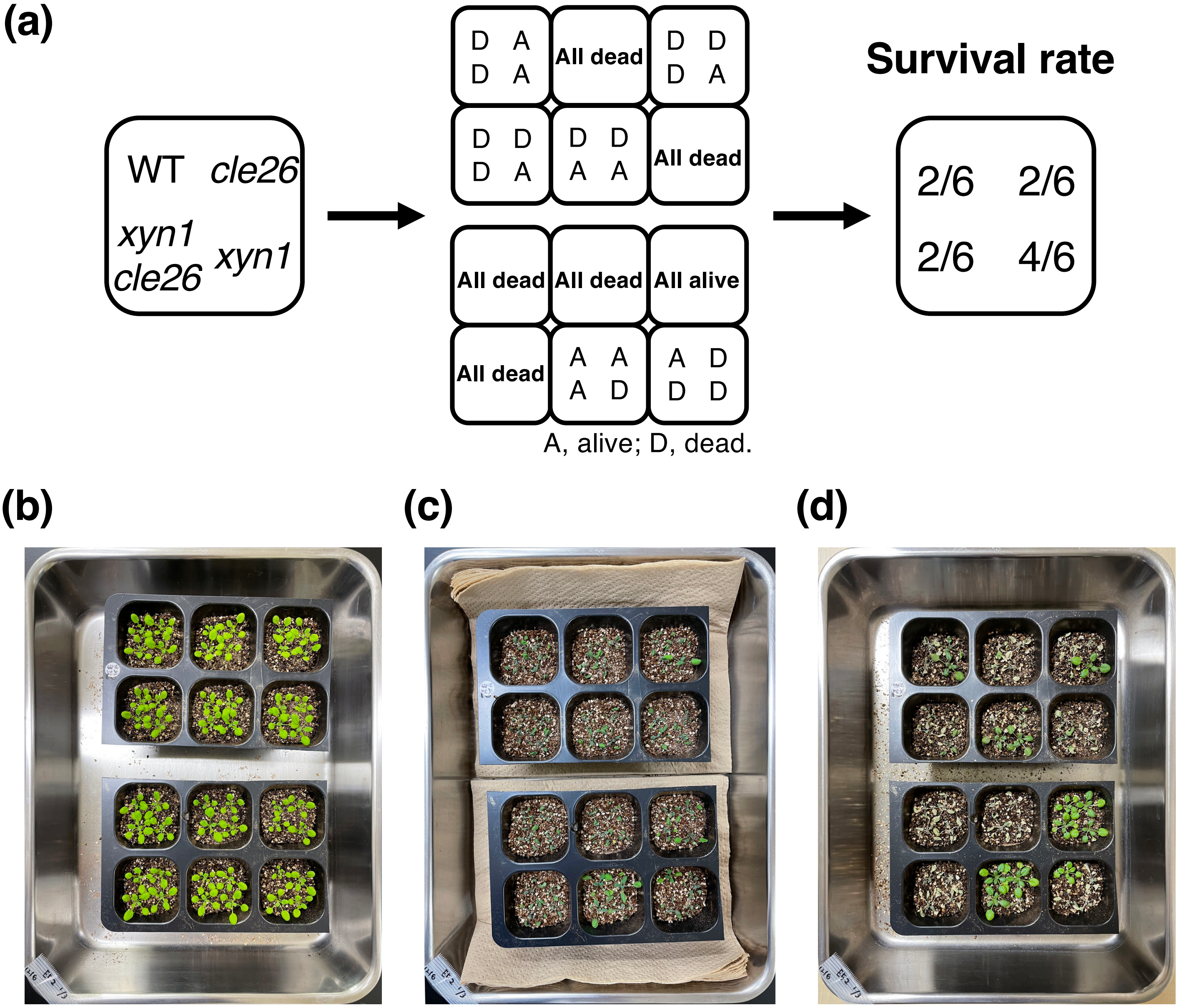

Fig. S1. Drought survival test

Plants with approximately six leaves (or four leaves for reproducing the drought-sensitive phenotype of the *cle25* mutant) at relative humidity (RH) > 45% were subjected to drought treatment. Each pot contained plants of different genotypes (a, b). First, plants were dehydrated on paper towels twice, and incubated under normal growth conditions for 1 d. Then, plants were incubated at RH of 20–40% for several days until most plants were wilted (c). Subsequently, the plants were rehydrated for 3 days (d). Because the dehydration treatment did not seem to affect each pot evenly, and was either too severe or too weak for some pots at any given time point, the pots with all dead or all alive plants were excluded, and the survival rate of plants was determined based on the remaining plants (a, d). As an exception, all pots were included in the comparison of the untreated and pretreated wild-type plants (Figs. 1i, S1c). To conduct the drought pretreatment, plants with approximately four leaves were subjected to dehydration for 2 d and then rehydrated.

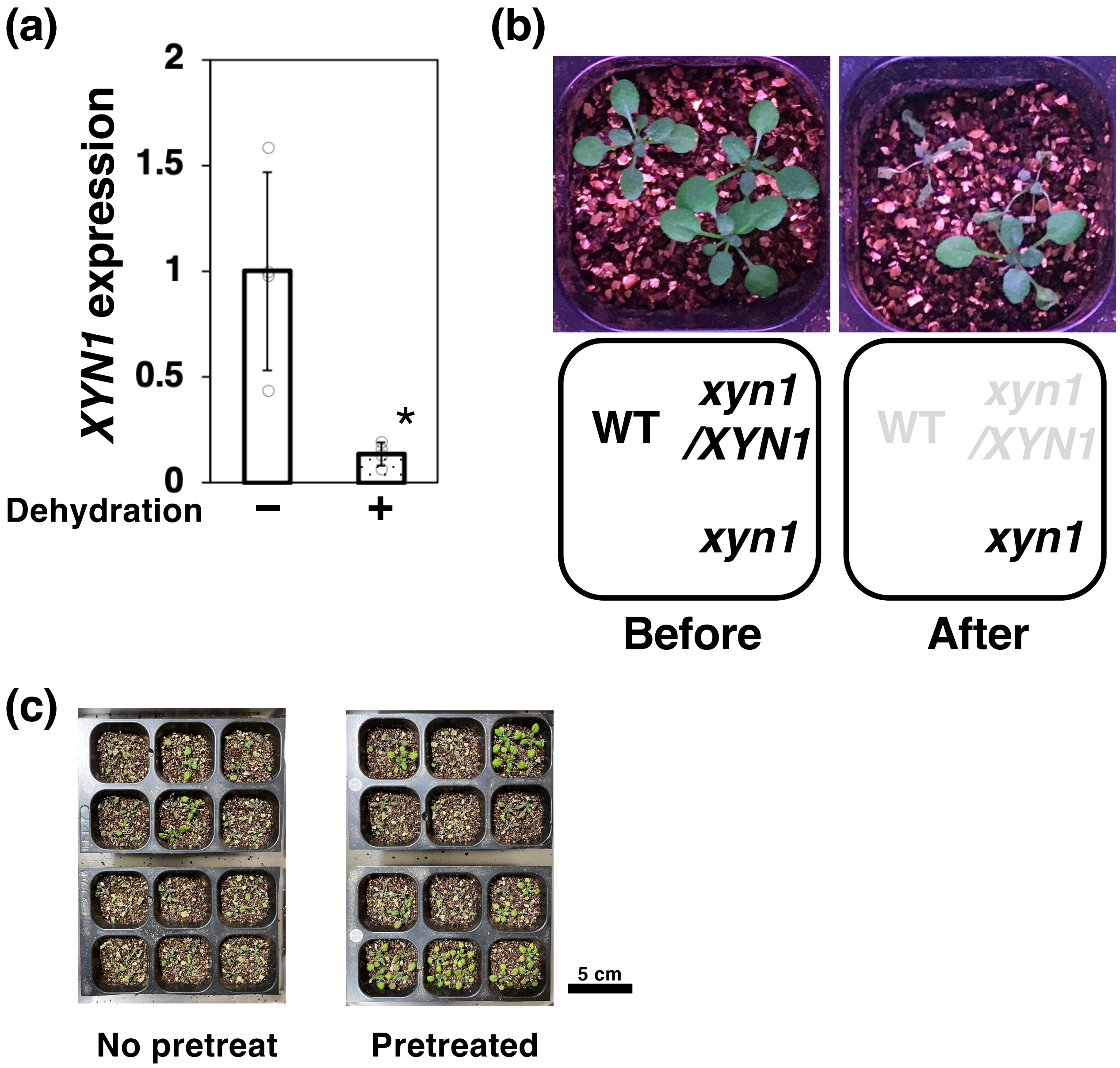

Fig. S2. *XYN1* expression and *XYN1*-associated drought resistance in *Arabidopsis thaliana*.

(a) *XYN1* expression upon dehydration. Plants grown on a conventional agar medium for 2 weeks were subjected to dehydration for 3 h. Samples with or without dehydration were examined by quantitative PCR. Values are presented as the mean ± SD ($n = 4$ [open circles]). Welch's t-test indicates a significant difference ($P < 0.05$).

(b) Drought resistance phenotype of the *xyn1* mutant plants, which were able to survive through the drought treatment compared with wild-type (WT) and *xyn1/XYN1* complementation plants.

(c) Drought resistance phenotype of the pretreated WT plants. In the pretreatment, WT plants at the four-leaf stage were dehydrated for 2 d, rehydrated, and recovered for 3 d. WT plants with or without the pretreatment were dehydrated until almost all the non-pretreated WT plants withered. Images were taken after the second rehydration for 3 d.

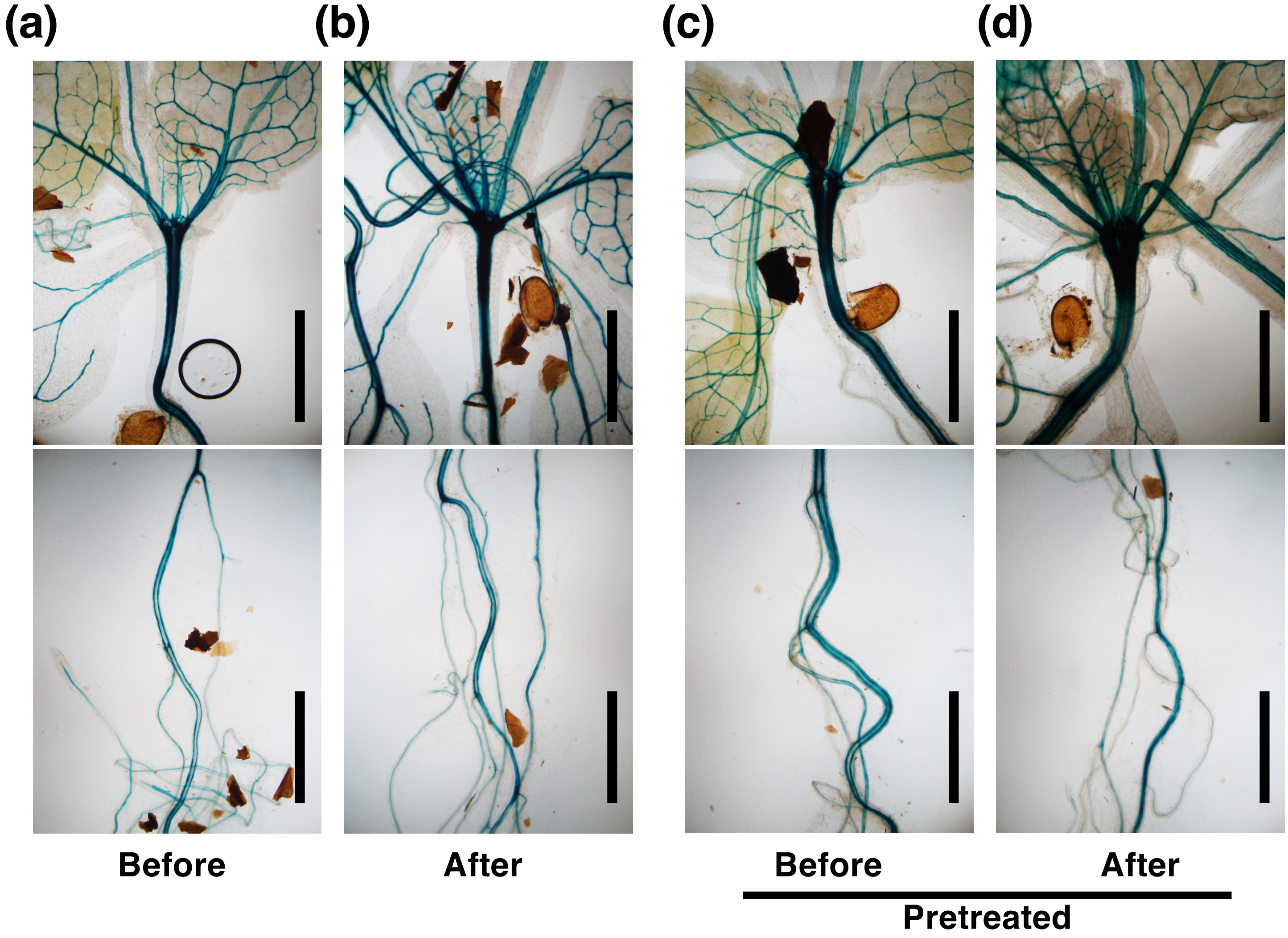

Fig. S3. *CLE26*-mediated drought resistance in *Arabidopsis*.

(a–d) *CLE26* expression upon dehydration. Prolonged (1 d) X-Gluc staining of plants harboring *pCLE26::GUS* before (a, c) and after dehydration for 1 d (b, d), with (c, d) or without the pretreatment (a, b). Close-up views of hypocotyls with juvenile leaves (upper panels) and roots close to the hypocotyls (lower panels). Bars, 1 mm. No clear changes in *CLE26* transcriptional activity were observed in response to dehydration.

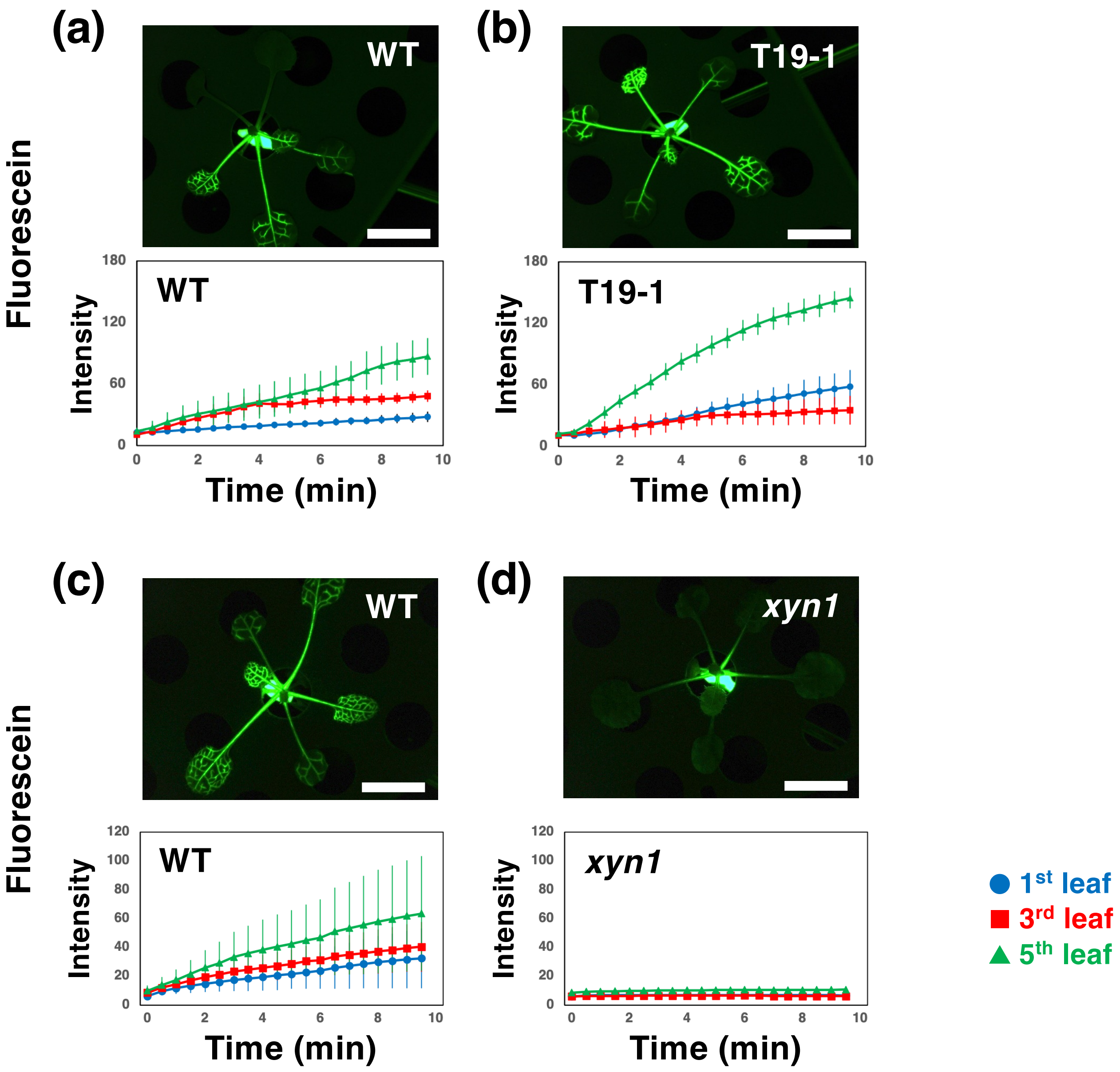

Fig. S4. *XYN1*-associated distribution of fluorescein in *Arabidopsis* at 10 min.

(a, b) Fluorescein signal in the wild-type (WT) and T19 plants.

(c, d) Fluorescein signal in WT and the *xyn1* mutant plants.

Upper panels show the fluorescence images of the indicated genotypes at 10 min after the application of fluorescein to the cut ends of hypocotyls. Images were taken from the top of plants with six leaves as described in (8). Scale bars, 5 mm.

Lower panels show the time-lapse analysis of fluorescein accumulation in leaves. Changes in fluorescence intensity (arbitrary unit) in the first (blue circles), third (red squares), and fifth (green triangles) leaves are shown at the indicated time points after the peptide application. Leaves are numbered from adult to young. Values are presented as the mean of three observations ± standard error of mean (SEM; $n = 3$).

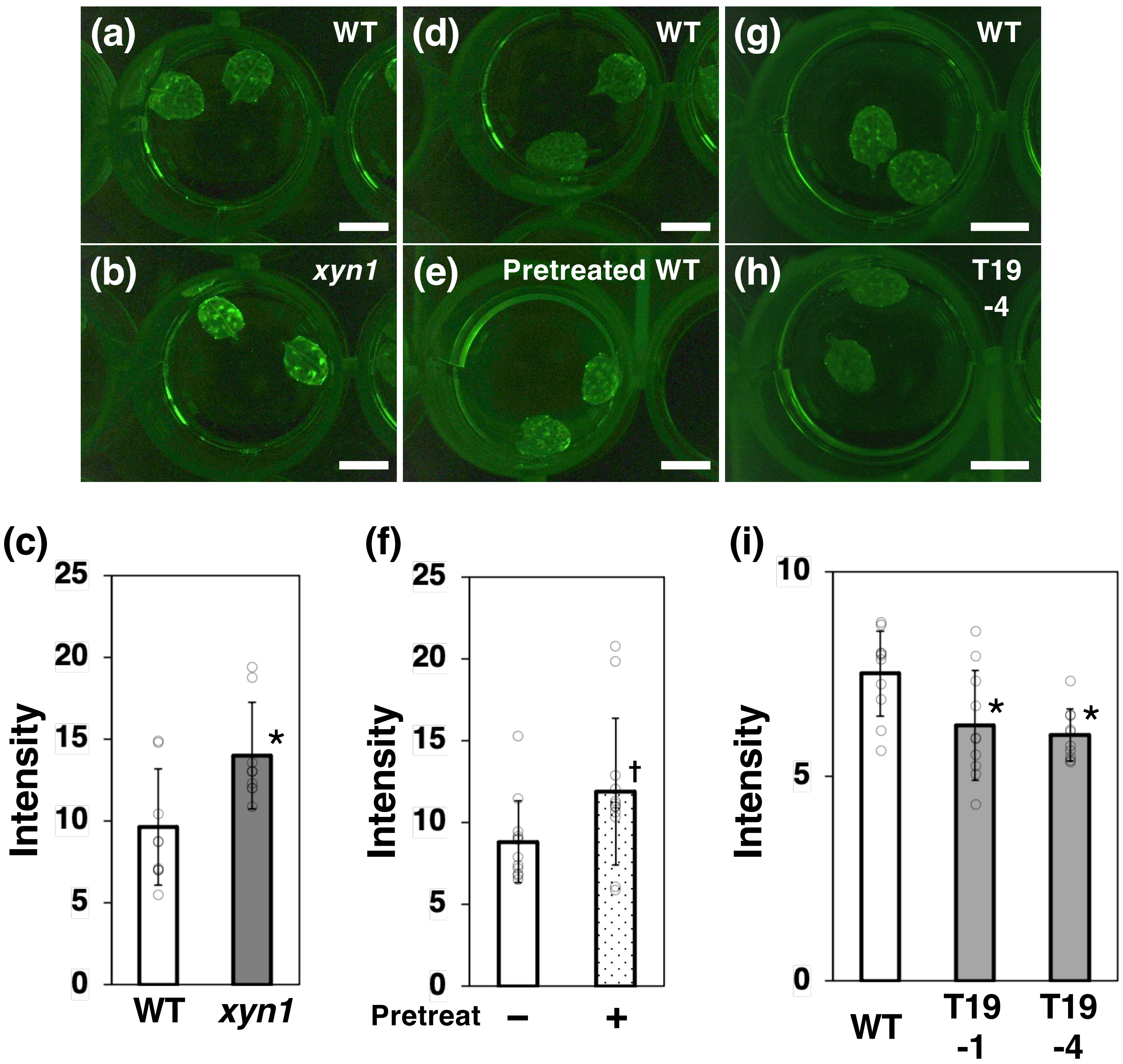

Fig. S5. *XYN1*-associated distribution of the fluorescent-labeled CLE26 peptide in detached leaves at high pH.

Fluorescence images of pairs of the third and fourth leaves, and quantification of fluorescence intensity (arbitrary unit) in these leaves. At 10 min after the application of FAM-labeled CLE26 peptide (CLE26F) to the cut end of hypocotyls, the leaves were detached and incubated in 0.07 M sodium phosphate solution (pH 9) for 10 min prior to observation. Scale bars, 5 mm.

(a–c) CLE26F fluorescence in the wild-type (WT) and the *xyn1* mutant plants. Values are presented as the mean ± SD ($n = 8$ [open circles]). Welch's *t*-test indicates a significant difference ($^{*}P < 0.05$).

(d–f) CLE26F fluorescence in WT with or without the drought pretreatment. Values are presented as the mean ± SD ($n = 12$ [open circles]). Welch's *t*-test indicates a possible difference ($^{\dagger}P = 0.052$).

(g–i) CLE26F fluorescence in WT and T19 plants. Values are presented as the mean ± SD ($n = 8$ [open circles]). Dunnett's test indicates significant differences compared with the WT ($^{*}P < 0.05$).

Table S1 Primers used in this study.

Target	Forward primer	Reverse primer
**Genomic *XYN1*** **for complementation**	caccAAATCTCTAAACCAATGCCAATTTAAG ↑ ***XYN1* upstream start**	CTGCATCATAACACCAACAACATCA ↑ ***XYN1* downstream end**
**3 kb upstream of *XYN1*** **for pXYN1::GUS**	caccAAATCTCTAAACCAATGCCAATTTAAG ↑ ***XYN1* upstream start**	tacaggacgtaacatGCTTTCTCTTGATTTCTAACATAACAA GUS start ↑ ↑ ***XYN1* upstream end**
**3 kb downstream of *XYN1*** **for pXYN1::GUS**	ggaggcaaacaatgaTTGTGACGCTTGTACATGTGATAAT GUS end ↑ ↑ ***XYN1* downstream start**	CTGCATCATAACACCAACAACATCA ↑ ***XYN1* downstream end**
**Coding sequence of GUS** **for pXYN1::GUS or pCLE26::GUS**	atgttacgtcctgtagaaaccccaac	tcattgtttgcctccctgctgcg
**Genomic *CLE26*** **for complementation**	caccAGCTTAATCTGATTAATCAGCGAAATT ↑ ***CLE26* upstream start**	CATAAACTTTTAACCTTAAATGTAACTC ↑ ***CLE26* downstream end**
**3 kb upstream of *CLE26*** **for pCLE26::GUS**	caccAGCTTAATCTGATTAATCAGCGAAATT ↑ ***CLE26* upstream start**	tacaggacgtaacatGGTTTCTAGCCTTTGTGGATATGTT GUS start ↑ ↑ ***CLE26* upstream end**
**3 kb downstream of *CLE26*** **for pCLE26::GUS**	ggaggcaaacaatgaTATTTCATTAATATATAATAATATATGACATA GUS end ↑ ↑ ***CLE26* downstream start**	CATAAACTTTTAACCTTAAATGTAACTC ↑ ***CLE26* downstream end**
***UBQ10* transcript** **with Universal Probe #119**	gaagttcaatgtttcgtttcatgt	ggattatacaaggccccaaaa
***XYN1* transcript** **with Universal Probe #50**	catttgaaaagacaatgtgacaaga	ccaccgggaagctatttctta
***NCED3* transcript** **with Universal Probe #22**	tcgtcgtgatagggtcctg	ttctcgtcagactcgttgaaaa

Table S2 Synthetic peptides used in this study.

ID	Structure
CLE25	NH2-RKV(Hyp)NG(Hyp)DPIHN-COOH
CLE26	NH2-RKV(Hyp)RG(Hyp)DPIHN-COOH
CLE25F	NH2-R(Lys[5(6)FAM])V(Hyp)NG(Hyp)DPIHN-COOH
CLE26F	NH2-R(Lys[5(6)FAM])V(Hyp)RG(Hyp)DPIHN-COOH
